# Hearing the light: neural and perceptual encoding of optogenetic stimulation in the central auditory pathway

**DOI:** 10.1038/srep10319

**Published:** 2015-05-22

**Authors:** Wei Guo, Ariel E. Hight, Jenny X. Chen, Nathan C. Klapoetke, Kenneth E. Hancock, Barbara G. Shinn-Cunningham, Edward S. Boyden, Daniel J. Lee, Daniel B. Polley

**Affiliations:** 1 Eaton-Peabody Laboratories, Massachusetts Eye and Ear Infirmary, Boston MA 02114; 2 Center for Computational Neuroscience and Neural Technology, Boston University, Boston, Massachusetts 02215; 3 Program in Speech Hearing Bioscience and Technology, Harvard Medical School (HMS), Boston MA 02115; 4 New Pathway MD Program, HMS 02115; 5 The MIT Media Laboratory, Synthetic Neurobiology Group, Massachusetts Institute of Technology (MIT), Cambridge, Massachusetts, USA; 6 Department of Biological Engineering, MIT, Cambridge, Massachusetts, USA; 7 Department of Otology and Laryngology, HMS, Boston MA, 02114; 8 Department of Biomedical Engineering, Boston University 02215

## Abstract

Optogenetics provides a means to dissect the organization and function of neural circuits. Optogenetics also offers the translational promise of restoring sensation, enabling movement or supplanting abnormal activity patterns in pathological brain circuits. However, the inherent sluggishness of evoked photocurrents in conventional channelrhodopsins has hampered the development of optoprostheses that adequately mimic the rate and timing of natural spike patterning. Here, we explore the feasibility and limitations of a central auditory optoprosthesis by photoactivating mouse auditory midbrain neurons that either express channelrhodopsin-2 (ChR2) or Chronos, a channelrhodopsin with ultra-fast channel kinetics. Chronos-mediated spike fidelity surpassed ChR2 and natural acoustic stimulation to support a superior code for the detection and discrimination of rapid pulse trains. Interestingly, this midbrain coding advantage did not translate to a perceptual advantage, as behavioral detection of midbrain activation was equivalent with both opsins. Auditory cortex recordings revealed that the precisely synchronized midbrain responses had been converted to a simplified rate code that was indistinguishable between opsins and less robust overall than acoustic stimulation. These findings demonstrate the temporal coding benefits that can be realized with next-generation channelrhodopsins, but also highlight the challenge of inducing variegated patterns of forebrain spiking activity that support adaptive perception and behavior.

For individuals with profound peripheral nerve degeneration, the only available treatment avenue for sensory restoration lies in delivering patterned electrical stimulation at early stages of central sensory processing. Although central prostheses have been used in human patients for over 50 years, they generally provide only a rudimentary sensory awareness. For example, translating spatial forms in the visual field to retinotopically organized microstimulation of primary visual cortex provides an awareness of object location, but does not support form discrimination^1^,^2^. Similarly, hundreds of deaf individuals have gained an awareness of environmental sound through auditory brainstem or midbrain implants. However, discrimination of complex signals such as speech is generally quite poor^3^,^4^,^5^,^6^, with a few notable exceptions^7^,^8^.

Interestingly, prostheses that deliver patterned electrical stimulation to retinal ganglion or spiral ganglion processes generally provide superior outcomes than stimulation of low-level brain areas (for review see Ref. 9,10). The abrupt performance drop associated with prosthetic devices that stimulate the brain rather than peripheral nerves may arise from more demanding surgical placement and electrical signal processing; yet the foremost cause arguably lies in the enormous increase in the complexity of coding and processing within brain networks themselves. Brain circuit organization varies widely across regions but most share a common logic that includes interconnected afferent recipient neurons, interneurons, feedback neurons, projection neurons and a host of glial cells that modulate chemical and electrical neurotransmission. Whereas electrical microstimulation indiscriminately activates multiple elements of these circuits, the use of genetically encoded light-activated ion channels (i.e., optogenetics) could provide the means to pinpoint stimulation to specific nodes within these circuits (for review see Ref. 11,12).

A host of ethical, engineering, and biological hurdles stand between the current implementation of optogenetic technologies in basic science research and the targeted delivery of channelrhodopsins to specific cell types in human patients. One fundamental problem lies in the fact that light-activated photocurrents are sluggish due to inherently slow channel kinetics in channelrhodopsins, which reduces their ability to deliver long lasting, high-frequency neural stimulation (e.g., above 40 Hz,^13^,^14^). This limitation is particularly problematic for any attempt to reconstitute sound representations in early stages of the auditory pathway, where temporal modulations in acoustic signals are normally encoded with submillisecond precision at rates as high as several hundred hertz^15^,^16^,^17^. Because precise, sustained spike timing is essential for auditory feature encoding, faithful reconstruction of sound representations by optogenetic stimulation also requires an ability to induce precise, fast, and non-adapting patterns of spiking activity.

Recently, a channelrhodopsin nicknamed ‘Chronos’ was identified in the algal species *Stigeoclonium helveticum.* Using a combination of patch clamp recordings from cultured neurons and acute brain slices, Chronos was revealed to have the fastest channel kinetics described in any naturally occurring or genetically engineered channelrhodopsin^18^. The advent of Chronos inspired us to explore the feasibility of an optoprosthesis for the central auditory pathway. The present experiments explore the suitability of inducing actionable auditory percepts by activating early stations of the central auditory pathway with optogenetic stimulation. Our first aim was to determine whether the superior temporal fidelity of Chronos over conventional ChR2 could also be demonstrated *in vivo,* using the central nucleus of the inferior colliculus (ICc), a central midbrain hub for acoustic signal analysis, as a test bed. We then determined how midbrain temporal coding differences translated to perceptual salience by measuring behavioral detection of acoustic or laser pulses presented at various rates. As a final step, we addressed discrepancies between neural coding in midbrain and behavioral performance by documenting how high-fidelity temporal codes for optogenetic stimulation in the midbrain were transformed in ostensibly disadvantageous ways at the level of the auditory cortex. Collectively, these findings support the feasibility of single channel optoprosthetic implants for basic auditory awareness but underscore the need to develop more sophisticated approaches for multi-channel cell type-specific activation for encoding spectrotemporally complex signals such as speech.

## Results

**Figure 1:**
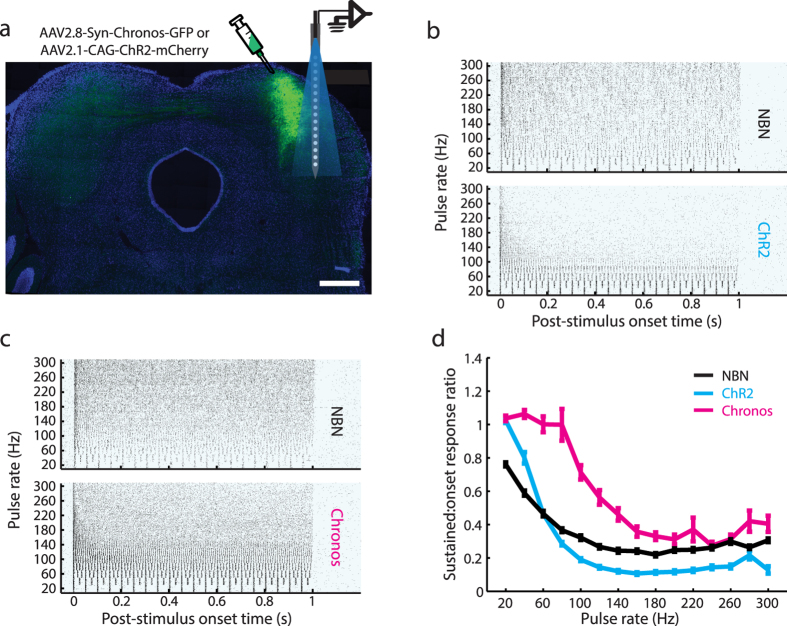
Chronos-mediated unit responses are more robust than ChR2 or natural acoustic stimulation. **** (**a**) Schematic of optrode recording probe relative to Chronos-GFP expression in a coronal section counterstained with DAPI. GFP expression levels are high in the central nucleus near the cartoon injection site with fainter staining in commissural axons that ramify in the contralateral ICc. Scale bar = 0.5 mm. (**b**,**c**) Rastergrams from individual recording sites stimulated either with 1 s trains of narrowband noise (NBN) bursts or pulsed blue light. (**d**) Firing rate adaptation quantified as the ratio between spikes evoked by the first pulse over the mean of all subsequent pulses. Error bars = sem.

Temporal variations in the sound pressure envelope provide the dominant physical cue for speech intelligibility^20^. To understand more about the accuracy and limitations of temporal coding with optogenetic stimulation of the central auditory pathways, we characterized the temporal response fidelity of midbrain neurons activated by trains of narrow-band noise (NBN) bursts or pulses of light at presentation rates ranging from 20–300 Hz. In these experiments, extracellular recordings of multiunit activity in the ICc were made several weeks after the same brain area was infected with a viral construct encoding either ChR2 or Chronos (Fig. 1a). In response to natural acoustic stimulation delivered to the contralateral ear, ICc neurons synchronize action potential timing to NBN pulse rates as high as several hundred Hz, with gradually adapting non-synchronized responses observed at higher rates (Fig. 1b). In ChR2 expressing regions (ChR2^+^), laser stimulation induced robust synchronization at low pulse rates (<50 Hz) and strongly adapting, non-synchronized responses at higher rates. By contrast, Chronos^+^ neurons could be entrained by a substantially wider range of photostimulation rates, with more sustained, non-synchronized activity observed even for the highest pulse rates tested (Fig. 1c). To quantify firing rate adaptation, we calculated the spike rate ratio between the first and the rest of the stimulus pulses for optogenetic versus acoustic stimulation. We found that adaptation increased with pulse rate for all modes of activation (*Repeated-measures ANOVA, N* = *388, df* = *14, F* = *365.4, p* = *2.9 × 10*^*−6*^) but was more pronounced overall for ChR2 compared to NBN (*Mixed-design ANOVA; main effect for stimulus type; N* = *117/160, df* = *1, F* = *22.8, p* = *2.9 × 10*^*−6*^). The opposite relationship was noted in Chronos^+^ neurons, in that adaptation was significantly more pronounced for acoustic pulse trains than optical pulse trains (*Mixed-design ANOVA; main effect for stimulus type; N* = *111/160, df* = *1, F* = *56.3, p* = *9.0 × 10*^*−13*^, Fig. 1d).

**Figure 2:**
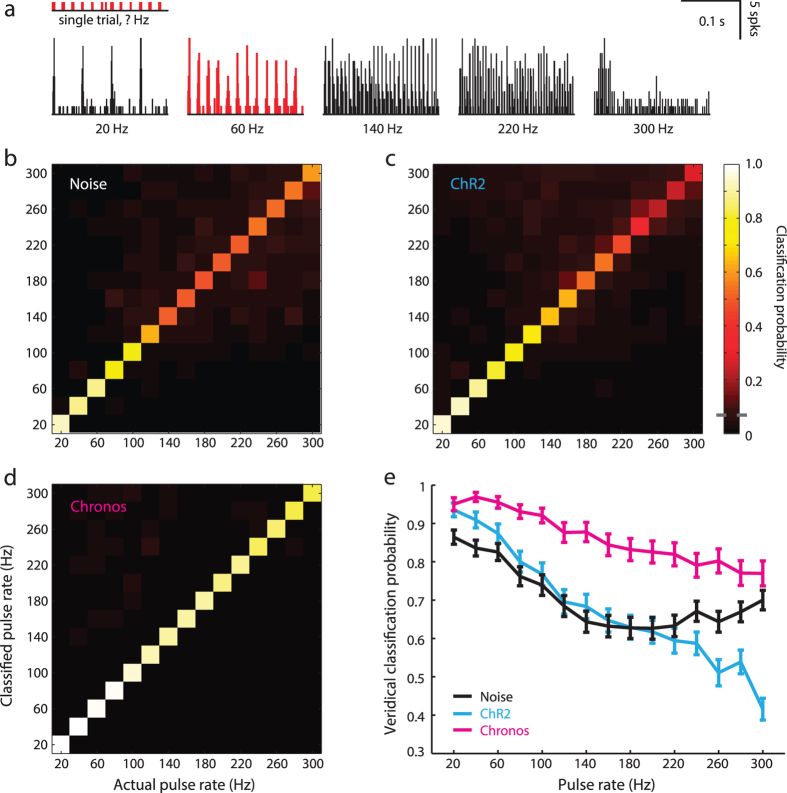
Chronos supports a superior neural code for stimulation rate. **** (**a–d**) A PSTH-based approach for single trial pulse rate classification from individual recording sites. (**a**) Representational templates for each pulse rate are established from 10 randomly selected trials (bottom row) and the remaining 10 trial are then individually assigned to the PSTH template that provides the closest match. Confusion matrices from representative single recording sites illustrate that classification is fairly accurate at low pulse rates but trails off for higher rates with narrowband noise (**b**) and ChR2 (**c**) but remains high across the full range of rates tested with Chronos (**d**). (**e**) Mean probability of veridical classification (i.e., the upward central diagonal from the confusion matrices) for each stimulation type. Error bars = sem.

To determine how the various firing rate profiles evoked by acoustic and optogenetic stimuli translated into a robust neural code for pulse rate, we used a PSTH-based classifier model to infer the parent stimulus rate from spike trains collected on individual trials. To the extent that each pulse rate was encoded by a distinct and reliable pattern of spiking, the classifier should correctly identify the pulse rate of the parent stimulus on an individual trial basis (Fig. 2a). The resultant confusion matrices computed from individual recording sites display the probability of veridical stimulus classification on the diagonal with classification errors appearing on either side. Qualitatively, all three types of activation support a robust neural code at low pulse rates, yet direct photoactivation via Chronos is the only approach that supports a robust code for higher pulse rates (Fig. 2b–d). Looking across all recording sites, we found a significantly higher probability of correct stimulus rate classification with Chronos compared to either ChR2 or NBN (*Mixed-design ANOVA; main effect for injection type; Chronos vs. ChR2: N* = *117/160, df* = *1, F* = *30.1, p* = *9.0 × 10*^*−13*^*, Chronos vs. NBN: N* = *111/160, df* = *1, F* = *21.3, p* = *6.0 × 10*^*−6*^*, both significant after the Bonferroni correction for 3 comparisons*) but no significant difference between ChR2 and NBN (*Mixed-design ANOVA; main effect for stimulus type; N* = *117/160, df* = *1, F* = *0.44, p* = *0.5, not significant after the Bonferroni correction for 3 comparisons*; Fig. 2e).

**Figure 3:**
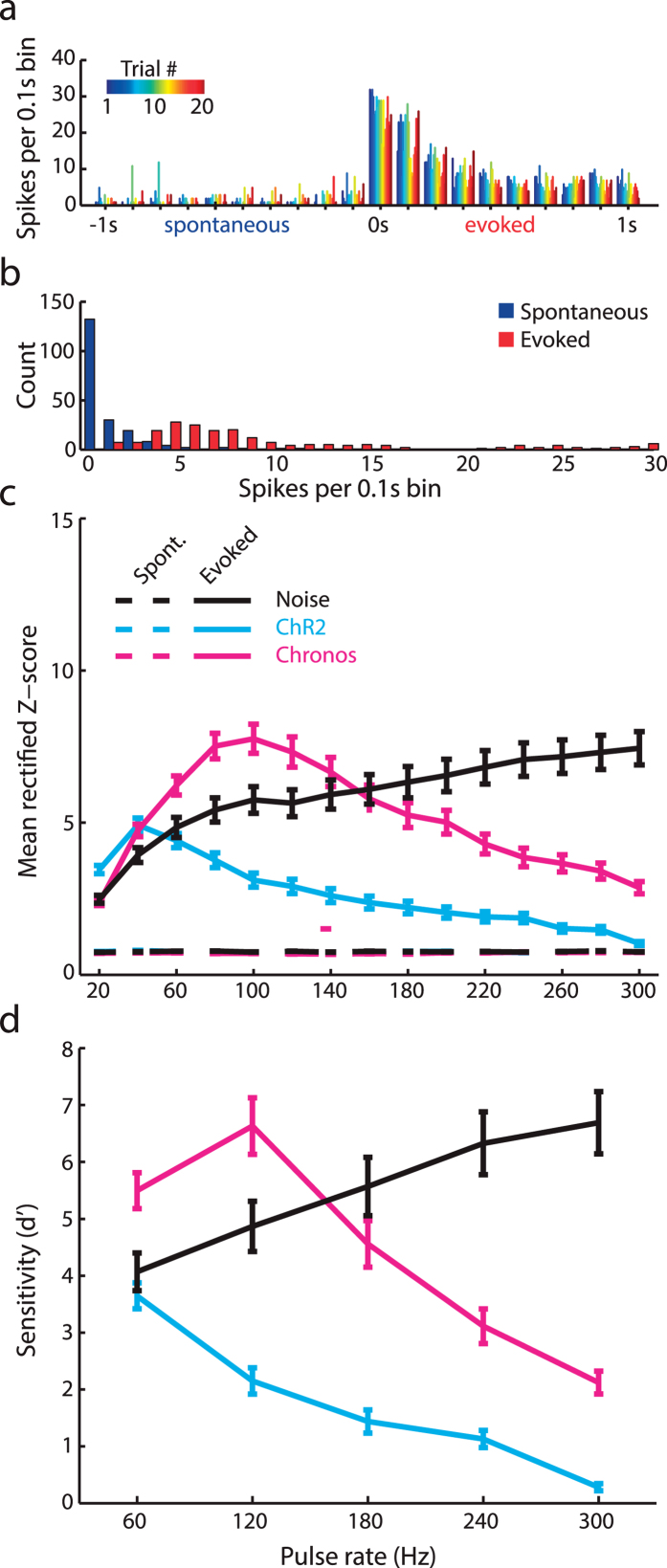
Chronos-mediated spiking provides a superior basis for pulse rate detection in the midbrain. **** (**a**) Firing rates from individual trials within a 2 s period surrounding the onset of photostimulation in a Chronos^+^ mouse is plotted in 0.1 s bins. (**b**) The distribution of single trial firing rates from each bin falling within the spontaneous (−1 to 0 s) and evoked (0 to 1 s) periods, derived from the results in panel A. (**c**) For all three forms of stimulation, rates from both periods are then converted into z-scores and the absolute values used as rectified z-scores, where more positive values represent deviations in firing rate that could support a neural code for detection. (**d**) the separation between the spontaneous and evoked z-score distributions is expressed with the d’ metric to quantify the utility of a rate code for detecting sound or photostimulation.

The previous analyses characterize the ability of midbrain neurons to discriminate between different pulse rates but leave open the more basic question of whether and how different methods of stimulation might support a more rudimentary code for simple detection of a stimulus. We addressed this point by counting the number of spikes in each 0.1 s bin for each trial (Fig. 3a) and comparing the overall distribution of spikes during the spontaneous and evoked periods (Fig. 3b). Spike counts from the spontaneous and evoked periods of the PSTH were then converted into z-scores and rectified (because any deviation from a baseline firing rate – be it an increase or decrease – could potentially drive detection), such that more positive z-scores represented a more salient neural cue for detection (Fig. 3c). We then computed the sensitivity index, d’, as the separation between the firing rate distributions evoked by the pulse train (hits) versus activity occurring during the pre-stimulus period (false positives) in units of standard deviations. We observed that both NBN and Chronos provided a salient cue for detection while the strong firing rate adaptation found with ChR2 stimulation interfered with a robust midbrain code for sound detection, particularly at high stimulation rates (*Mixed-design ANOVA; between subjects factor; ChR2 vs. NBN: N* = *117/160, df* = *1, F* = *44.3, p* = *1.5 × 10*^*−10*^*, ChR2 vs. Chronos: N* = *111/160, df* = *1, F* = *56.2, p* = *1.5 × 10*^*−12*^, both *significant after the Bonferroni correction for 3 comparisons*; Fig. 3d). The clear differences in neural d’ that emerged from our midbrain neurophysiology experiments inspired a set of core hypotheses related to the perceptual salience of central auditory activation via sound versus direct photoactivation of midbrain neurons: *i)* behavioral detection of NBN should be robust and relatively insensitive to pulse rate; *ii)* behavioral detection of optogenetic stimulation should decrease at higher pulse rates; *iii*) Chronos should support an enhanced behavioral detection of midbrain photostimulation compared to ChR2.

**Figure 4:**
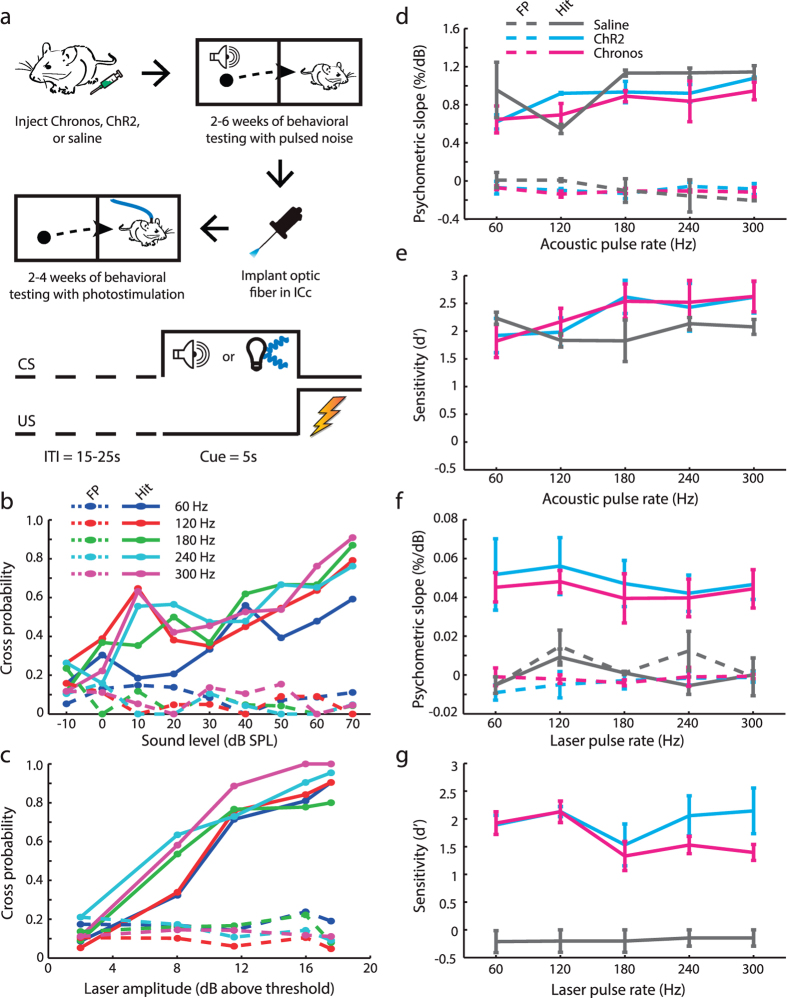
Behavioral detection of midbrain photostimulation is similar across pulse rates and opsin types. **** (**a**) Detection abilities were tested with an avoidance paradigm where mice crossed from one side of a shuttlebox to another to avoid receiving an electric shock (top). Crossing during the 5 s cue period was scored as a hit while crossing during an equivalent 5 s period during the intertrial interval (ITI) was scored as a false positive (FP, bottom). (**b**,**c**) Hit and FP probabilities are plotted as function of sound level (**b**) and laser amplitude (**c**) across all pulse rates for a single, representative mouse. (**d–g**) The slope of the linear fit applied to each psychometric function obtained with acoustic (**d**) and laser (**f**) stimulation provides an objective readout for salience of pulse rate detection. Behavioral d’ is calculated from the acoustic (**e**) and laser (**g**) stimulation level that supports a 50% hit probability for each pulse rate.

To address these hypotheses, mice were trained to report detection of pulse trains delivered via with acoustic stimulation or optogenetic stimulation of the midbrain. Shortly after receiving unilateral ICc injections of viral constructs or saline, mice were trained and tested in an auditory avoidance task. Specifically, NBN pulse trains of variable level and rate were presented, whereupon mice crossed sides of a shuttlebox to avoid receiving a foot shock (Fig. 4a). Once psychometric functions were obtained for all acoustic pulse rates (Fig. 4b), an optic fiber assembly was implanted into the previously injected ICc and the behavioral procedure was repeated with photostimulation in lieu of acoustic stimulation (Fig. 4c). Increasing stimulation amplitude was associated with a monotonic increase in behavioral detection probability (Fig. 4b,c). The slope of the growth function for each pulse rate could be estimated from a linear fit of the data, which provided a behavioral proxy for stimulus salience.

As predicted from the high neural d’ values for acoustic stimulation at all pulse rates (Fig. 3d), detection slopes were similarly steep across all NBN pulse rates. Psychometric detection slopes did not vary between injection types, but were affected by pulse rate (*Mixed-design ANOVA; main effect for injection type: N* = *3/3/2, df* = *2, F* = *1.1, p* = *0.40; main effect for pulse rate: N* = *3/3/2, df* = *4, F* = *6.6, p* = *1.5 × 10*^*−3*^, Fig. 4d). The d’ sensitivity index was then computed at a fixed sensation level (50 dB above the detection threshold, which usually generates hit rates around 70%). This analysis supported the conclusion that detection of acoustic pulse trains was robust for all injection types, and modestly affected by pulse rate (*Mixed-design ANOVA; main effect for injection type: N* = *3/3/2, df* = *2, F* = *0.32, p* = *0.74; main effect for pulse rate: N* = *3/3/2, df* = *4, F* = *7.0, p* = *1.0 × 10*^*−3*^, Fig. 4e).

Upon switching the detection stimulus from sound to light we found that Chr2^+^ and Chronos^+^ mice immediately generalized across stimulation modalities (Movie S1). To our surprise, however, Chronos^+^ and ChR2^+^ mice exhibited similar detection slopes (*Mixed-design ANOVA; main effect for injection type: N* = *3/3/2, df* = *2, F* = *0.13, p* = *0.73;* Fig. 4f) and d’ values (*Mixed-design ANOVA; main effect for injection type: N* = *3/3/2, df* = *2, F* = *0.05, p* = *0.83;* Fig. 4g) that did not vary as a function of pulse rate (*Mixed-design ANOVA; main effect for pulse rate; slope: N = 3/3/2, df = 4, F = 1.4, p = 0.32; d’: N = 3/3/2, df = 4, F = 0.83, p = 0.52*). Detection was at chance in saline-injected mice, confirming that detection of photostimulation trains could not be ascribed to visual detection of reflected laser light (*Paired t-test between hit rates and FP rates, N = 10, p = 0.34*). Similar results were obtained when behavioral crossing latency was substituted for crossing probability (Fig. S1). Thus, behavioral findings in channelrhodopsin-expressing mice were essentially opposite to the predictions based on our ICc recordings, from which we surmised that Chronos^+^ mice would be more sensitive than ChR2^+^ and that perceptual salience be reduced at higher pulse rates.

**Figure 5:**
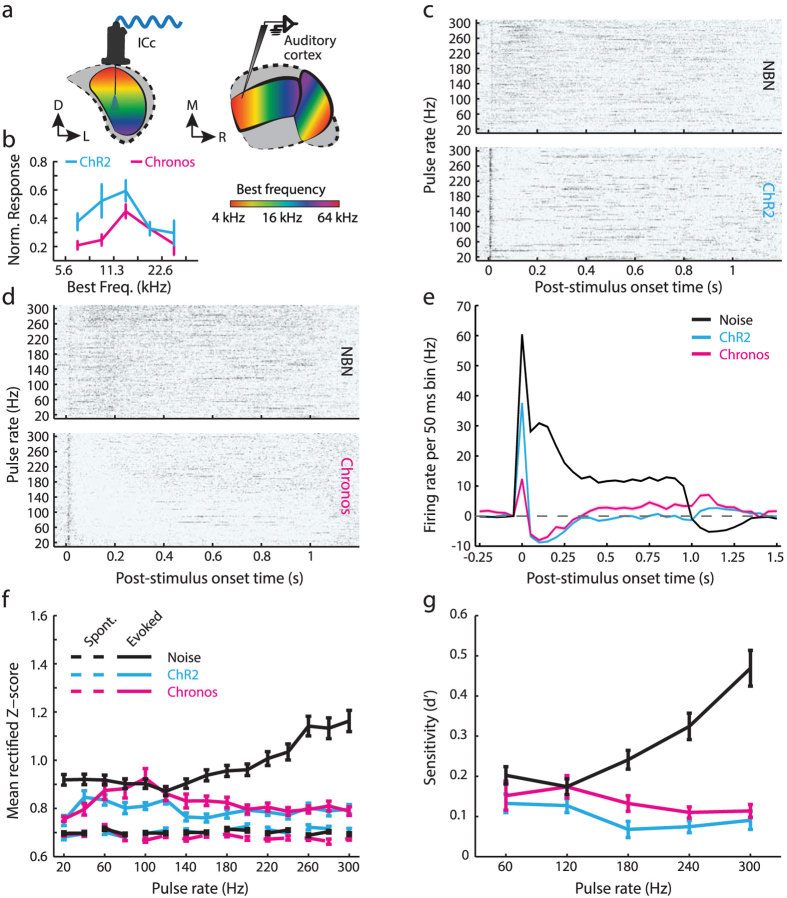
Auditory cortex encoding of midbrain photostimulation matches behavior detection, not ICc neural detection. **** (**a**) AC recordings were made following the completion of behavioral testing using a laser power corresponding to a 0.7 hit probability for each pulse rate. The optical fiber tip was positioned to photostimulate a mid-frequency region of the ICc tonotopic map, which (**b**) activated a corresponding region of the AC tonotopic map. (**c**,**d**) Rastergrams from individual recording sites depict stimulation with 1 s trains of NBN bursts or pulsed blue light. (**e**) PSTHs averaged across all pulse rates reveal brief, weak onset responses followed by suppression with optogenetic stimulation and comparatively robust, less adapting responses evoked by NBN. (**f–g**) Rectified z-scores and the resultant d’ index were calculated for each pulse rate and stimulation type as described previously for ICc recordings in Fig. 3.

One potential explanation for these discrepant findings stems from the possibility that robust midbrain neural codes for the discrimination (Fig. 2) and detection (Fig. 3) have been reformatted, diminished, or lost altogether upon reaching higher stations of the auditory CNS, where neural activity is more intimately linked to perception^21^,^22^,^23^,^24^,^25^,^26^,^27^,^28^. For example, neural activity in the auditory cortex (AC) might be organized in a manner that more closely resembles behavioral detection functions than midbrain activity. We addressed this hypothesis by recording activity from the core fields of AC (the primary auditory cortex and the anterior auditory field) in the same mice that were tested behaviorally. For the sake of comparison to the ICc data, we recorded multiunit activity evoked by acoustic pulse trains and midbrain optogenetic stimulation under anesthesia with similar stimulation and recording protocols (Fig. 5a).

Optic fiber tip implantation and virus injections were made at 0.35 mm and 0.7 mm below the dorsal surface of the inferior colliculus, respectively. These depths approximately correspond to a best frequency range of 10–22 kHz within the ICc tonotopic map, respectively (Fig. 5a**, S2**). By sampling optogenetically-evoked spiking across the AC tonotopic map, we confirmed that feedforward activation from the midbrain was greatest within zones of the AC with similar best frequencies (Two-way *ANOVA; main effect for BF; N = 42/45, df = 4, F = 3.97, p = 0.54 *×* 10*^*−3*^; Fig. 5b). Aside from this topographic correspondence, the downstream effects of optogenetic stimulation measured in the AC bore little resemblance to the direct activation of midbrain neurons. Whereas acoustic stimulation evoked non-synchronized, partially adapting responses in AC, photostimulating midbrain ChR2^+^ (Fig. 5c) or Chronos^+^(Fig. 5d) ICc neurons evoked brief, non-synchronized onset responses (<50 ms) followed by suppression and a rapid return to baseline (Fig. 5e). As such, sound-evoked responses in AC were significantly greater overall than either mode of direct midbrain activation (One-way *ANOVA; NBN vs. ChR2: N = 136/64, df = 1, F = 1540.67, p < 1 *×* 10*^*−196*^*; NBN vs. Chronos: N = 136/72, df = 1, F = 902.82, p < 1 *×* 10*^*−196*^).

To determine if AC neural detection across pulse rates more closely resembled midbrain or behavioral detection functions, we set the laser power for each pulse rate to be 12 dB above the detection threshold for each pulse rate, where hit rates were generally greater than 70%. We found that AC neural detection of optogenetic stimulation was fairly constant across pulse rates and was similarly robust for ChR2 and Chronos (but lower than for NBN), both in terms of both the z-scores separating the evoked and spontaneous periods (Fig. 5f) as well as the derivative d’ values (Fig. 5g). For NBN inputs, d’ increased significantly across pulse rates in a similar fashion to ICc recordings (Repeated measures ANOVA, N = 160, df = 4, F = 69.6, p = 0,Fig. 3d) and was significantly greater overall than for either mode of optogenetic stimulation (*Mixed-design ANOVA; main effect for injection type;* NBN vs. ChR2: N = 136/48, df = 1, F = 17.7, p = 4.1* × *10^−5^; NBN vs. Chronos: N = 136/72, F = 17.2, p = 4.9* × *10^−5^). In contrast with ICc results, neural detection of midbrain photostimulation did not significantly differ across opsin type (*Mixed-design ANOVA; main effect for opsin type: N = 48/72, df = 1, F = 0.64, p = 0.42*). Although there was a significant effect of pulse rate (Repeated measures ANOVA; ChR2: N = 48, df = 4, F = 4.7, p = 1.1* × *10^−3^; Chronos: N = 72, df = 4, F = 8.2, p = 3.0* × *10^−5^), the absolute change in AC detection with either type of photostimulation was modest.

To determine if AC spiking represented the temporal features of midbrain photostimulation at the population level in a manner that could not be appreciated from an analysis of single recording sites, we applied a population based approach to calculate the z-scores of cortical neural responses, where for a given stimulus, its population neural z-score is the highest z-score out of all the recorded sites. However, even at the level of a distributed cortical population code, midbrain photostimulation was similarly poor for both opsins (*Mixed-design ANOVA; main effect for opsin type: N = 3/3, df = 1, F = 0.05, p = 0.84,* Fig. S2). Thus, the cortical encoding of optogenetic pulse trains did not support the second and third hypotheses that arose from our midbrain findings, but closely resembled – and likely enabled – similar observations made at the level of behavior.

## Discussion

Auditory prostheses have provided over 300,000 deaf individuals with an awareness of environmental sound and an ability to comprehend speech in quiet backgrounds. In this regard, the auditory prosthesis is a triumph of modern biomedical engineering. Speech intelligibility in quiet has steadily improved among cochlear implant recipients since the device came into common use approximately 30 years ago. Average cochlear implant users who are fitted with modern electrodes and electronic processing strategies correctly comprehend approximately 58% of monosyllabic words presented in silence^29^. However, the inter-subject variability in speech comprehension is enormous, even after controlling for mitigating variables such as onset and duration of deafness. For example, individuals implanted at the same clinic by the same surgeon using identical hardware, exhibit speech recognition scores ranging from 0–100%^30^. Moreover, such devices rarely allow users to extract intelligible information from complex, degraded acoustic signals such as speech in noise. Speech recognition with auditory brainstem or midbrain implants is even worse, with users reporting 35% (n=60) and 0% (n=3) improvements in comprehension, respectively, for measures of speech performance^6^,^8^.

The present project was motivated by a desire to explore alternative ways to provide an actionable sound percept through direct brain stimulation. We took advantage of a recently described channelrhodopsin, Chronos, that supports non-adapting, high-fidelity encoding of rapid light pulses that fall within the range of pulse rates used in auditory brainstem implant processors (SPEAK processing strategy, 250 pulses/s, Cochlear Clinical Guidance Document 2010). Direct comparison of acoustic versus optical activation in ChR2^+^ or Chronos^+^ ICc neurons demonstrated that Chronos supported a superior encoding of temporal pulse trains, in terms of firing rate adaptation (Fig. 1), temporal coding (Fig. 2) and the overall salience of neural responses at high stimulation rates (Fig. 3). These performance advantages were not realized when the downstream effects of midbrain photostimulation were assessed behaviorally (Fig. 4) or through AC recordings (Fig. 5).

We found that midbrain photostimulation induced weak cortical onset responses followed by firing rate suppression across a broad range of stimulation frequencies, consistent with previous descriptions of cortical response suppression upon electrical stimulation of the inferior colliculus in animals^31^,^32^. Thus, a spatiotemporally coherent barrage of afferent activity might be ill-suited to engage the distributed coding capacities of cortical neurons. One possible explanation lies in the observation that fast-spiking parvalbumin^+^ inhibitory interneurons, which are known to rapidly ‘quench’ excitatory responses in primary sensory cortex^33^,^34^, have broader frequency tuning and lower response thresholds than putative pyramidal neurons^35^, but see Ref. 36. Thus, simultaneously exciting a relatively broad region of the subcortical tonotopic map might preferentially engage cortical inhibition, thereby preempting robust, sustained responses in cortical excitatory neurons, which are more responsive to afferent regimes characterized by spatially differentiated patterns of inputs organized on multiple, nested time scales^37^,^38^,^39^,^40^,^41^,^42^.

An alternative explanation holds that cortical networks do indeed encode temporal information contained within the photoactivated midbrain efferent signals, but that the nature of the code has been reformatted from a temporal scheme that is evident in individual midbrain recording sites to a spatially distributed, rate-based scheme that can only be appreciated when documented at the level of coordinated activity an ensemble of cortical neurons. Indeed, internally or externally generated inputs to auditory cortex are processed by locally organized subnetworks of cortical neurons^43^,^44^,^45^,^46^ that feature highly stereotyped spatiotemporal dynamics^47^ that generate inter-neuronal correlations, which may^48^ or may not^49^ augment AC coding capabilities. Although our approach did not permit the direct visualization of cellular ensembles in the cortex (Fig. S3), behavioral detection exhibited a similarly weak correspondence to midbrain encoding functions, suggesting that even if temporal features were encoded that could not be observed with our neurophysiological approach they are not being translated into behavioral detection abilities.

If temporal coding deficiencies can be attributed to the stimulation strategy and not to the readout, how could one design a central sensory prosthesis better suited to the encoding preferences of higher brain networks so as to provide a more robust percept? We suggest that the core deficiency lies in the inability to selectively activate the projection neurons of low-level sensory brain areas with spatially differentiated inputs. Whereas a single cochlear implant channel activates a relatively homogenous population of cochleotopically restricted afferent nerve fibers, direct stimulation of the auditory brainstem or midbrain activates a heterogeneous and spatially undifferentiated population of neurons and glial cells. This deficiency is insurmountable with electrical stimulation strategies but could be addressed through further development of an optogenetics-based prosthesis.

What would be the defining features of a future optoprosthesis? Ideally, the channelrhodopsin could be selectively expressed in primary output neurons at the earliest stage of central auditory processing (e.g. globular and spherical bushy cells, multipolar, and fusiform neurons^50^,^51^) . Cell-type specific expression is trivial to realize in laboratory animals, where Cre-*loxP* transgenic recombination systems used in conjunction with Cre-dependent viruses ensure that transcription of viral constructs are limited to the cell type of interest^52^. An alternative approach that might be more feasible in humans would be to design viral constructs with promoter sequences that are transcribed at far higher levels in the cell type of interest than neighboring cells. In this manner, transcription of the channelrhodopsin DNA could be ‘steered’ into particular cell types within the cochlear nucleus. This approach has been used with some success in the cerebral cortex, where viral constructs packaged with the CAMKIIa promoter are expressed at far greater levels in pyramidal neurons due to natural variations in expression of this gene between different cell types^53^. *In situ* hybridization studies of the cochlear nucleus or its avian homolog reveal striking differences in mRNA levels between cell types, which lends provisional support to further study of transcriptional differences between principal neurons and other cell types of the cochlear nucleus (e.g. mGluR1α in globular bushy cells^54^; VGlut2 in fusiform neurons^55^).

The challenge of creating spatially differentiated input patterns could be tackled through an engineering approach to develop bundles of low-power, miniature-scale independent illumination systems or a biological approach to engineer channelrhodopsins that are sensitive to restricted, non-overlapping wavelengths^18^. With this latter approach, the brainstem could be ‘seeded’ with constructs encoding distinct channelrhodopsins that were packaged in viral serotypes selected to provide highly focal infection zones. Alternatively, a single construct encoding multiple channelrhodopsins with a self-initiated transcriptional suppressor system might be used to drive stochastic expression of a single channelrhodopsin from the cassette, similar to the approach used for multi-colored fluorescent protein expression in ‘Autobow’ mice^56^.

Provided a minimal level of spatiotemporally differentiated activity, ample time for adjustment, and a cellular milieu that supports an acceptable degree of plasticity, higher levels of the brain can support a remarkable constellation of perceptual operations despite a distorted or impoverished input signal. In classic examples, humans and other animals rapidly adapt to visual goggles that dramatically shift the visual field^57^ or to temporary manipulations of the outer ear that grossly distort dichotic cues that underlie sound localization^58^. Perhaps even more impressively, postlingually deafened cochlear implant users can map the massively altered and degraded patterns of electrical stimulation onto prior neural representations of acoustic speech sounds. Further, they can rapidly recover or exceed baseline speech recognition when temporal stimulation strategies are radically changed^59^ or can achieve a fused pitch percept from bilateral implants that stimulate grossly mismatched cochlear positions^60^. Thus, whatever the form optoprosthesis development may take, future recipients, surgeons, and scientists can take solace from the observation that the prosthetic device need not provide a perfect signal, but rather one that is just good enough to fuel the remarkable pattern recognition abilities of the brain.

## Methods

All procedures were approved by the Massachusetts Eye and Ear Infirmary Animal Care and Use Committee and followed the guidelines established by the National Institute of Health for the care and use of laboratory animals. A total of 20 male CBA/CaJ mice were used in this study (10 for *in vivo* ICc recordings; 10 for behavioral assessments and *in vivo* Actx recordings).

### Virus injection

Adult CBA/CaJ mice aged 8–10 weeks were sedated with isoflurane (5% in oxygen), then anesthetized with ketamine (100 mg/kg) and xylazine (10 mg/kg). A surgical plane of anesthesia was maintained with supplements of ketamine (50 mg/kg) as needed. Throughout the procedure, the animal’s body temperature was kept at around 36.5 °C with a homeothermic blanket system. After numbing the scalp with lidocaine (0.5%), an incision was made along the midline, exposing the skull around the lambdoid suture. A small craniotomy (0.2* × *0.2 mm, with the medial-rostral corner positioned at 0.4 mm lateral and 0.1 mm caudal to lambda) was made with a scalpel to expose the right inferior colliculus. The dura mater was left intact. Electrophysiological recordings were made to identify the location of the central nucleus (ICc) before virus injection (See acute electrophysiology in the IC). Glass capillary pipettes were pulled and back filled with mineral oil before loading with virus. A motorized stereotaxic injector (Stoelting Co.) was used to inject 0.3–0.5 μl of either AAV-CAG-ChR2-mCherrry or AAV-Synapsin-Chronos-GFP into the right ICc of the mouse approximately 700 μm below the brain surface with an injection rate of 0.05 μl/min. The pipette was left in place for an additional 10 minutes before withdrawal. The craniotomy was covered with high viscosity silicon oil, and the scalp was sutured back. Mice were allowed to recover for at least 48 hours before behavioral training with NBN and at least 3 weeks before detection was measured with optogenetic stimulation.

### Acute electrophysiology in the IC

The surgical procedure was similar as described in the previous section. Mice were anesthetized and a craniotomy performed over the right IC. Single-shank multi-channel silicon optrodes (NeuroNexus Technologies) were used to deliver laser pulses and record neural activity (sampled at 24 kHz, digitized at 32 bit, and then band-pass filtered between 300 to 5000 Hz with second-order Butterworth filters). Multiunit spike events on each channel were time stamped at threshold crossing (4.5 s.d. above a 10 s running average of the baseline activity, SpikePac, Tucker-Davis Technologies). All recordings were performed in a double-walled sound-attenuating chamber. The ICc was identified according to the dorsal-ventral low-high tonotopic organization as defined by a pseudorandom series of pure tone pips (4–64 kHz in 0.1 octave steps, 0–60 dB SPL in 5 dB steps, 50 ms duration with 5 ms cosine ramps at the onsets and offsets, 500 ms inter-trial intervals) presented to the contralateral ear with a custom-built, calibrated in-ear acoustic system.

Laser pulses (473 nm, 1 ms pulse width, 1 s total duration, LaserGlow Co.) were presented at various rates (20 to 300 Hz, 20 Hz steps) to the IC via the optic fiber on the optrode, which was positioned 0.2 mm above the topmost recording site. To avoid potential contamination through photoelectric artifacts, threshold crossings during the laser pulse were disregarded. Laser powers were selected to generate suprathreshold responses in the infected tissue on a case-by-case basis, and were generally in the range of 5 to 7 mW. For the sake of comparison narrowband noise bursts (filtered from broadband noise stimuli using fourth-order Butterworth filters, 20 kHz center frequency and 0.25 octave bandwidth, 1 ms duration, 60 dB SPL) were presented at the same rates via the contralateral in-ear acoustic system. Laser and acoustic stimulation were presented in a pseudorandom order and repeated 20 times each.

### Optic fiber implantation

Following 2–6 weeks of behavioral testing with acoustic stimuli, mice were anesthetized with ketamine and xylazine, as described previously. An implantable 4 mm optic fiber assembly (NeuroNexus NNC fiber) was advanced 0.35 mm into the ICc along the previous injection site. The implant was then securely cemented on the skull (C&B Metabond). Mice were allowed to recover for at least 48 hours prior to the continuation of behavioral testing.

### Acute electrophysiology in the auditory cortex

Following previously described procedures^19^, ChR2^+^ and Chronos^+^ mice were anesthetized with ketamine and xylazine, and a craniotomy was made over the right auditory cortex. The exposed dura was covered with high viscosity silicon oil. Extracellular recordings of multiunit activity were made with tungsten electrodes (FHC Co.) positioned in the middle cortical layers. Acoustic stimuli were delivered to the contralateral ear via a calibrated in-ear acoustic system. Laser stimuli were delivered through the implanted optic fiber in the ipsilateral ICc. Acoustic and laser stimulus parameters were identical to the approach used for ICc recordings. Since animals at this stage had all completed behavioral training and assessment, the peak amplitude used for acoustic and laser stimulation was set to a suprathreshold level according to the corresponding behavioral from each mouse (60 dB SPL and 12 dB above the laser detection threshold, respectively).

### Behavioral testing

Behavioral training was carried out in an acoustically transparent enclosure (8* *×* *6* *×* *12 inch, L* *×* *W* *×* *H) bisected into two virtual zones resting atop electrified flooring (8 pole scrambled shocker, Coulbourn Instruments). Mouse position was tracked with a commercial PC webcam. Auditory stimuli were delivered through a free-field speaker positioned above the apparatus to provide a relatively homogenous sound field (Tucker-Davis Technologies). Mice were given at least five minutes to acclimate to the apparatus before each day of testing. Naïve mice were initially shaped to cross between zones of the chamber to terminate a foot shock (60 Hz, 0.5–1 mA, according to the minimally effective intensity for each mouse). With conditioned crossing behavior established, mice were then trained to associate sound (white noise, 5 s duration, 5 ms cosine ramps, 70 dB SPL) with foot shock initiated 5 s later. Crossing within the 5 s window was scored as a hit and the foot shock was avoided. Foot shock was initiated if the mouse failed to cross within the 5 s period (a miss) and was terminated upon crossing sides or 10 s, whichever occurred first. Once the hit rate stabilized at ≥ 70%, white noise was replaced with the narrow-band noise bursts and training continued until crossing behavior stabilized again. Psychometric functions were acquired by documenting the hit probability at different sound levels (−10 to 70 dB SPL in 10 dB steps) and pulse rates (60–300 Hz in 60 Hz steps). Stimuli were presented in a pseudo random fashion and repeated at least 15 times each. Inter-trial intervals were randomly drawn from a uniform distribution between 30 to 40 seconds. False positives were calculated as animal’s crossing probability during a 5 s window halfway through the inter-trial period. Typically, each animal performed 60 to 100 trials per day, 5 to 6 days per week.

For behavioral experiments involving detection of laser pulse trains rather than acoustic pulse trains, the implanted midbrain assembly was tethered to the laser with a patch cable. Mice were given approximately 20 minutes to acclimate to tethering before conditioned crossing behavior was initially reestablished with broadband noise stimulus. Once the hit probability was comparable to that documented without tethering, the acoustic stimulus was replace by laser stimuli (1 ms laser pulses, 60 to 300 Hz in 60 Hz steps) without any additional behavioral shaping. Due to the variability of sensitivity introduced by injection volume and expression level of the opsins, the range of laser intensity tested was adjusted on a case-by-case basis for each animal to generate a range of subthreshold to suprathreshold behavioral responses. In all other respects, stimulus design and task organization were identical to the acoustic version of the task.

### Histology

Animals were deeply anesthetized with ketamine and prepared for transcardial perfusion with 4% formalin solution. The brains were extracted and post-fixed in 4% formalin at room temperature for an additional 12 hours before transfer to 30% sucrose solution. Brain sections (60 μm thick) were counterstained with DAPI (Life Technologies). The position and size of the infection zone was inferred through visualization of the fluorescent label with a conventional epifluorescence microscope (Zeiss).

### Data analysis

Firing rate adaptation was quantified by calculating the ratio of the spike count to the first pulse divided by the average spike count to all remaining pulses within the 1 s period. To quantify the temporal fidelity of sound or laser evoked activities, a template-based classifier model was used. For any given recording site, half the trials of responses to all pulse rates were used to build peristimulus time histogram (PSTH) based templates; the other half of the trials were used as test cases. Test trials were compared with the templates by calculating their cross correlation coefficients. The decoded pulse rate for a test trial was the pulse rate behind the most similar template (highest cross correlation coefficient). The decoding accuracy for all rates was calculated and averaged across recording sites. Pulse train detectability was quantified by dividing the PSTH into 100 ms bins and calculating the firing rates for each bin within the spontaneous and evoked periods on a single trial basis. For any given bin, its detectability was quantified as the rectified z-score of its spike count with respect to the baseline distribution. The difference between mean z-scores from the spontaneous and evoked periods for each trial provided the basis for calculating d’. In the population version of this analysis, the neural detectability for a given stimulus was calculated as the highest detectability provided by any of the recorded sites from an animal.

### Statistical analyses

All statistical analyses were performed in Matlab (Mathworks). Repeated measures ANOVAs were used to compare neural or behavioral measurements over dependent variables such as pulse rate or sound intensity in the same group of animals. When comparing measurements across different groups of animals, mixed-designed ANOVAs were used, and the main effects were reported. Multiple comparisons were corrected with the Bonferroni method.

## Author Contributions

W.G. and D.B.P. proposed and designed the experiments. W.G., A.E.H. and J.X.C. performed the experiments. K.E.H. provided essential help in hardware and software setup. N.C.K. and E.S.B. provided critical reagents. W.G., A.E.H. and D.B.P. wrote the manuscript. J.X.C., N.C.K. B.G.S.-C., E.S.B. and D.J.L. revised the manuscript. B.G.S.-C., D.J.L. and D.B.P. supervised the project.

## Additional Information

**How to cite this article**: Guo, W. *et al.* Hearing the light: neural and perceptual encoding of optogenetic stimulation in the central auditory pathway. *Sci. Rep.* **5**, 10319; doi: 10.1038/srep10319 (2015).

## Supplementary Material

Supplementary Information

Supplementary Movie 1
