# Supplementary material for: Hearing the light: neural and perceptual encoding of optogenetic stimulation in the central auditory pathway
Source: Sci Rep. 2015 May 22;5:10319. doi: 10.1038/srep10319 (PMC4441320; doi:10.1038/srep10319)
Supplement: Supplementary Information [file srep10319-s1.pdf]

Hearing the light: neural and perceptual encoding of optogenetic stimulation in the central auditory pathway

Wei Guo[1,2*], Ariel E Hight[1,3], Jenny X Chen[1,4], Nathan C Klapoetke[5,6], Kenneth E Hancock[1,7], Barbara G Shinn-Cunningham[2,8], Edward S Boyden[5,6], Daniel J Lee[1,7], and Daniel B Polley[1,2,7*]

1- Eaton-Peabody Laboratories, Massachusetts Eye and Ear Infirmary, Boston MA 02114
2- Center for Computational Neuroscience and Neural Technology, Boston University, Boston, Massachusetts 02215
3- Program in Speech Hearing Bioscience and Technology, Harvard Medical School (HMS) Boston MA 02115
4- New Pathway MD Program, HMS 02115
5- The MIT Media Laboratory, Synthetic Neurobiology Group, Massachusetts Institute of Technology (MIT), Cambridge, Massachusetts, USA.
6- Department of Biological Engineering, MIT, Cambridge, Massachusetts, USA
7- Department of Otology and Laryngology, HMS, Boston MA, 02114
8- Department of Biomedical Engineering, Boston University 02215

*Contact:

Wei Guo – weiguo@bu.edu
Prof. Daniel Polley - daniel_polley@meei.harvard.edu

Eaton-Peabody Laboratories, Massachusetts Eye and Ear Infirmary
243 Charles St. Boston, MA 02114

Supplementary Materials

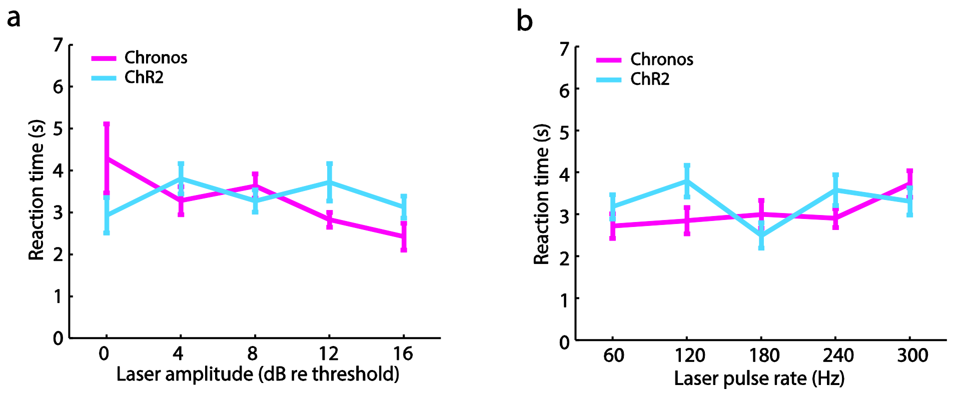

Fig. S1. Animals' reaction time towards the laser stimulation. Behavioral crossing latency, rather than crossing probability, is plotted as a function of laser pulse amplitude (**a**) and rate (**b**).

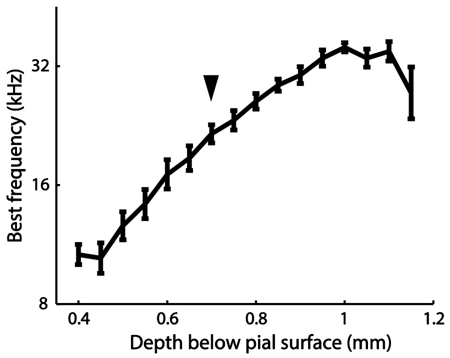

Fig. S2. The ICc is tonotopically organized along the dorsal-ventral axis. The best frequency of recorded sites (N = 10 penetrations, 16 sites per penetration) in the ICc are plotted as a function of depth, revealing a tonotopic organization. The arrow indicates the depth of virus injection.

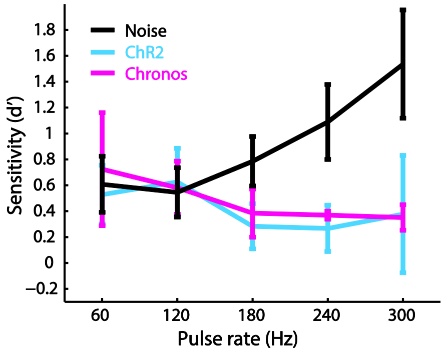

Fig. S3. The performance of population-based detection matches the performance of single-site-based detection. The sensitivity indices (d') for stimuli with different pulse rates are measured with a population decoding scheme, where the population d' is quantified as the highest single-site-based d' out of all the recorded sites from a given animal.

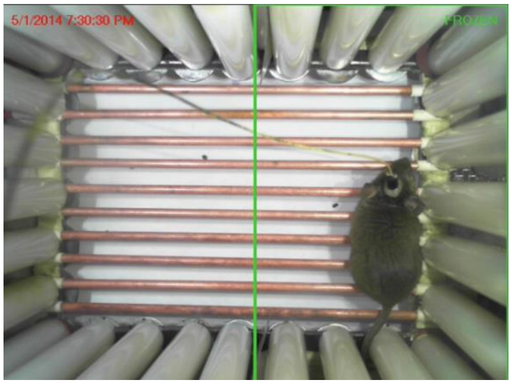

Movie S1. Animals showed generalized avoidance behavior across stimulus types. All animals with either ChR2 or Chronos expressed in the ICc would show avoidance behavior towards laser stimulation highly similar to that of sound stimulation.
